# Reinforcing Mechanisms of Coir Fibers in Light-Weight Aggregate Concrete

**DOI:** 10.3390/ma14030699

**Published:** 2021-02-02

**Authors:** Xiaoxiao Zhang, Leo Pel, Florent Gauvin, David Smeulders

**Affiliations:** 1 Department of Applied Physics, Transport in Permeable Media, Eindhoven University of Technology, P.O. Box 513, 5600 MB Eindhoven, The Netherlands; l.pel@tue.nl; 2 Department of Built Environment, Building Materials, Eindhoven University of Technology, P.O. Box 513, 5600 MB Eindhoven, The Netherlands; F.Gauvin@tue.nl; 3 Department of Mechanical Engineering, Energy Technology, Eindhoven University of Technology, P.O. Box 513, 5600 MB Eindhoven, The Netherlands; d.m.j.smeulders@tue.nl

**Keywords:** natural fiber, coir fibers, hydration, shrinkage, internal curing, reinforcement, fiber treatment

## Abstract

Due to the requirement for developing more sustainable constructions, natural fibers from agricultural wastes, such as coir fibers, have been increasingly used as an alternative in concrete composites. However, the influence of coir fibers on the hydration and shrinkage of cement-based materials is not clear. In addition, limited information about the reinforcing mechanisms of coir fibers in concrete can be found. The goal of this research is to investigate the effects of coir fibers on the hydration reaction, microstructure, shrinkages, and mechanical properties of cement-based light-weight aggregate concrete (LWAC). Treatments on coir fibers, namely Ca(OH)_2_ and nano-silica impregnation, are applied to further improve LWAC. Results show that leachates from fibers acting as a delayed accelerator promote cement hydration, and entrained water by fibers facilitates cement hydration during the whole process. The drying shrinkage of LWAC is increased by adding fibers, while the autogenous shrinkage decreases. The strength and toughness of LWAC are enhanced with fibers. Finally, three reinforcement mechanisms of coir fibers in cement composites are discussed.

## 1. Introduction

Due to the requirement for high-rise, long-span, or functional buildings, light weight aggregate concrete (LWAC) has attracted increasing attention because of its unique advantages, for example, lower density, larger specific strength, and superior thermal insulation [1,2]. However, LWAC also has drawbacks such as low flexural strength and poor fracture toughness, which have restricted its wider applications [3]. These defects could be usually compensated by adding fibers such as steel or synthetic fibers [4,5]. Nevertheless, even though these fibers are found to positively influence the properties of LWAC, some correlatively negative effects should be addressed. For example, both the steel and synthetic fibers have both high material and energy consumption, and can lead to a substantial environmental footprint in their production processes [6]. Moreover, synthetic fibers can cause health risks (e.g., skin irritations or respiratory diseases) during the manufacturing and bring environmental burdens due to difficulty in disposing [7]. Some synthetic fibers like carbon fibers is not economical [8]. Additionally, steel fibers significantly increase the density of LWAC, especially when their fraction exceeds 1 vol.% [9].

Unlike artificial fibers, natural fibers (NFs) are eco-friendly, renewable, recyclable, and disposable, and have other advantages such as low cost, light-weight, and good mechanical properties [10,11]. Brittle materials reinforced with natural fibers exhibit equivalent mechanical performance as with synthetic fibers [12,13,14]. Thus, NFs are a good candidate to replace synthetic fibers to reinforce concrete with cost-effective and environmental benefits. Furthermore, due to the compatibility of NFs and LWAC, both have relatively light-weight and low mechanical properties, therefore, NFs are especially suitable for reinforcing LWAC. Among the various available NFs, coir fibers extracted from waste coconut husks are abundant, with approximately 500,000 tons of coir fibers produced annually worldwide [15]. As compared to the most used NFs such as flax or bamboo, coir fibers possess a comparable specific tensile strength and have higher toughness and flexibility [16]. Moreover, coir fibers can preserve good mechanical properties under aggressive conditions [17]. Some studies have been carried out to investigate the reinforcing effect of various contents and lengths of coir fibers on the mechanical performance of concrete. The results show that coir fibers significantly enhance the flexural strength, toughness, and impact resistance of concrete [18,19], and their optimum content ranges from 1 wt.%–3 wt.% (about 2 vol.%–12 vol.%) with length of 1–5 cm [20,21].

However, because of the main properties of NFs such as low resistance against the alkaline environment and also high water absorption, using coir fibers can lead to some issues into the cement matrix. Primarily, some constituents of coir fibers such as hemicellulose can be easily decomposed into various sugars under the alkaline environment in cement, which can affect the cement hydration kinetics [22,23]. Secondly, coir fibers are hydrophilic and thus their high moisture absorption can cause competitive water absorption with cement, thereby affecting the available water amount for cement hydration [24,25]. Saturated fibers can be used to take advantage of this feature, thus, extra water can be entrained in the cement system for internal curing [26]. Nevertheless, few previous studies of NFs as reinforcements in concrete show the effect of their leachates, indicating a delayed effect on cement hydration [27]. Furthermore, only several presented studies are related to the internal curing effect of macro saturated fibers [26,28].

In addition, the efficiency of fiber reinforcement principally depends on the fiber and matrix interfaces. Due to the heterogeneity of coir fibers and cement matrix, their bonding is relatively poor, and the Interfacial Transition Zones (ITZs) are commonly weak, leading to micro-cracks and degradation of mechanical properties. To improve the compatibility between NFs and cement, different pretreatments for NFs such as heat treatment, organic solvent, and alkali treatments are usually adopted [29,30]. These pretreatments generally aim to remove the unwanted constituents of NFs at their surface as well as to increase the relative cellulose content, since this latter is stable and is known as the strongest constituent of NFs. This results in a more homogeneous fiber surface, thus providing a better bonding with the matrix. Furthermore, they reduce the potential leachates that can affect the cement hydration [31]. However, these removed constituents are demonstrated in contributing to the flexibility of NFs, and consequently, fibers could lose their reinforcing effect after pretreating [32,33]. Therefore, improved treatment methods should be studied to further improve the overall properties of cement composites without deteriorating fibers.

Accordingly, the objective of this study is to investigate the reinforcement mechanisms involving leaching effect and internal curing of saturated coir fibers in LWA concrete. Moreover, to further promote the mechanical performance of concrete and to retain efficient properties of coir fibers, two treatment methods, namely Ca(OH)_2_ and nano-silica impregnation, have been adopted for coir fiber modification. The concrete is designed by adopting the modified Andreasen and Andersen (A&A) particle packing model to achieve a more compacted matrix, and LWAs made of waste glass are applied to develop a greener concrete. The influence of coir fibers with various contents and different treatments on hydration kinetics, microstructure, drying and autogenous shrinkages, and mechanical properties of cement composites are analyzed. Finally, the reinforcing mechanisms of coir fibers on cement-based light-weight aggregate concrete are proposed from three conceivable prospects, namely hydration acceleration, internal curing, and mechanical bridging.

## 2. Materials and Methods

### 2.1. Materials

**Figure 1:**
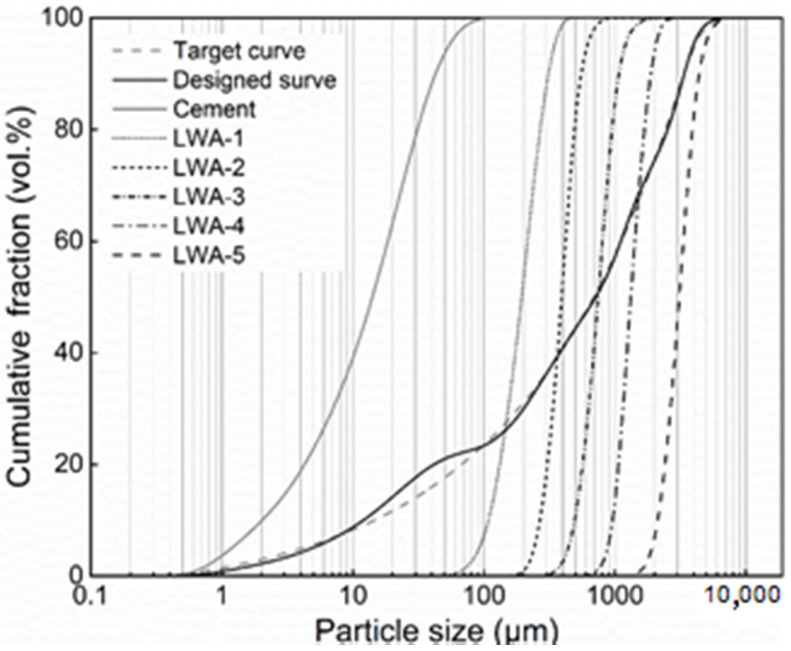
Particle size distributions of the cement and five light-weight aggregates (LWAs), and the resulting target curve and integral grading curve of the mixture.

The cement used is Ordinary Portland Cement (OPC) CEM I 52.5 R, supplied by HeidelbergCement (Heidelberg, The Netherlands). The Particle Size Distributions (PSDs) of cement are determined by Laser Particle Size Analyzer (LPSA) (Malvern Panalytical, Worcestershire, UK), shown in Figure 1.

**Figure 2:**
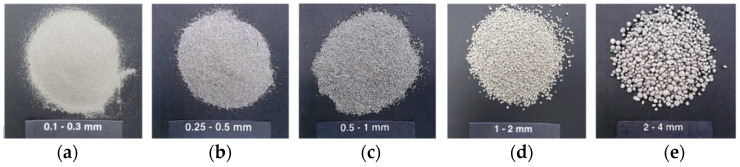
Appearance of LWAs: Expanded waste glass aggregates with 5 different diameters of (**a**) 0.1–0.3, (**b**) 0.25–0.5, (**c**) 0.5–1, (**d**) 1–2, and (**e**) 2–4 mm, respectively.

**Figure 3:**
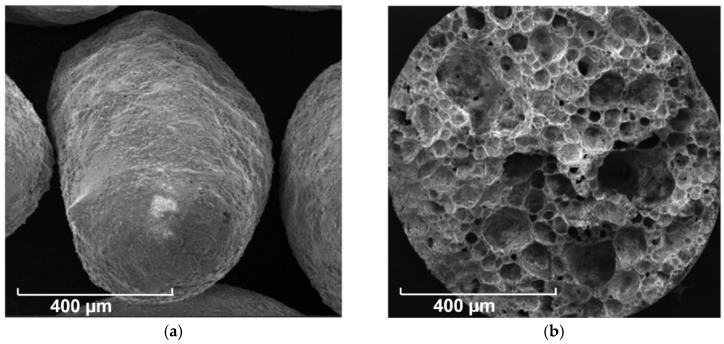
SEM pictures of outer surface and inner porous structure of LWAs: (**a**) LWA-3, (**b**) 0.5–1.0 mm.

The light-weight aggregates presented in Figure 2 are produced from recycled glass, supplied by Liaver Company (Ilmenaucity, Germany). The Particle Size Distributions of LWA is also shown in Figure 1. These LWAs have encapsulated cellular structures inside, but a closed external shell outside, resulting in their low water absorption. More information about the physical properties and chemical compositions of LWA is given in this research [34]. The SEM graphs of LWAs microstructure are shown in Figure 3.

**Figure 4:**
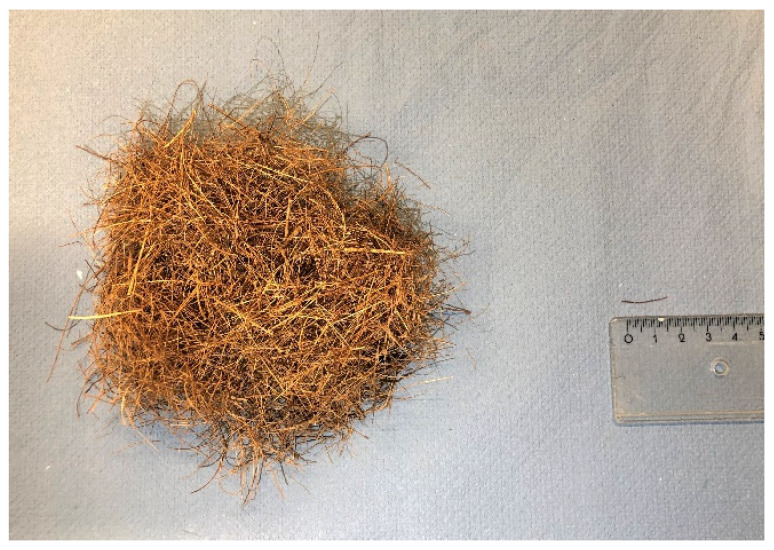
Appearance of coir fibers. The average length of coir fibers is about 15 mm.

Coir fibers are provided by the Wageningen University and Research (Wageningen, The Netherlands), shown in Figure 4. The physical and chemical properties of coir fibers are shown in Table 1. The bulk density is measured as the mass of coir fibers divided by their total bulk volume, and the specific density is tested by a Helium pycnometer (AccuPyc II 1340 Micromeritics, Veldhoven, The Netherlands) based on ASTM D1895-17 [35]. The bundles of coir fibers are cut into short fibers with the length of 15 mm. The average diameter of coir fibers is measured by Scanning Electron Microscopy (SEM). The average tensile strength of coir fibers is tested by single fiber tensile test (SFTT) according to ASTM D2343–17 [36]. The chemical components of coir fibers and leachates of coir fibers in distilled water are analyzed by a high-performance anion exchange chromatography (HPAEC). The detailed testing methods can be found in our previous study [23]. The leaching solution of coir fibers is mainly constituted of different monomeric sugars and a low amount of acid, given in Table 2.

### 2.2. Fiber Treatment

All coir fibers used in this study are saturated with water. Additionally, two treatments, namely Ca(OH)_2_ and nano-silica impregnation, are employed for absorbing and depositing calcium and silica to the surface and inside coir fibers. These treatments aim to obtain more hydration products on and inside of fibers, and thus improve the ITZs between fibers and cement. A saturated calcium hydroxide solution prepared from Ca(OH)_2_ (96% purity) and nano silica slurry with a concentration of 50% and an average diameter of 0.12 µm provided by Merck (Darmstadt, Germany) and AkzoNobel (Amsterdam, The Netherlands), respectively, are prepared for fiber impregnation. The volume ratios of both treating agents to coir fibers are controlled at 1.2:1. All coir fibers are firstly washed several times with water until the color of water become clear and next they are dried in the open air at a room temperature of 20 °C for 7 days. Subsequently, the dried fibers are immersed in distilled water, Ca(OH)_2_ solution and nano-silica solution for about 2 h, respectively. After that, coir fibers are filtrated to remove the liquids and dried with absorbent paper. Finally, fibers are sealed in containers for later use.

### 2.3. Mixture Design and Mixing Procedures

The mix proportions of LWAC are presented in Table 3. The mixtures of LWAC are designed by applying the modified Andreasen and Andersen (A&A) packing model to optimize the granular packing of all solid materials [38].

(1) $$P\left(D\right)=\frac{D^{q}-D_{\min}^{q}}{D_{\max}^{q}-D_{\min}^{q}}$$

where P(D) is the fraction of total solid materials with particle sizes lower than D (μm). D_min_ and D_max_ are the smallest and largest particle sizes (μm), which are 0.4 and 4000 μm, respectively. q is the distribution modulus. By optimizing q values, which determine the particle size proportions of concrete. A larger q results in a coarser mixture and a recommended q value is in the range of 0–0.4 [39,40]. In order to achieve a targeted strength as well as a low density of LWAC, a relatively large q is chosen as 0.35 for all mixtures. The PSDs of the target curve and the resulting integral grading curve (designed curve) of the mixture are shown in Figure 1.

To avoid segregating without deteriorating the workability of the mixes, a water/cement ratio is fixed at 0.4. The dosages of coir fibers are 0.5%, 1.5%, and 3% by weight of the concrete. Meanwhile, a polycarboxylic ether based superplasticizer (SP) is adopted to adjust the workability of concrete, and all slumps of concrete are controlled under about 140 mm following ASTM C230. According to the adopted fiber contents, the amounts of SP are 0.2%, 0.45%, and 0.8% by weight of the cement, to remedy the workability loss caused by adding fibers. For the reference mixture without fibers, the SP amount is used as 0.15%.

The mixing procedures are carried out as described below. Cement and LWAs are firstly put in a mixer for dry mixing about 1 min. Then around 75% of water is gradually added and mixed with the cement and LWAs for about 2 min, meanwhile coir fibers are consistently fed. Subsequently, the remaining water mixed with SP is added and mixed for an additional 2 min. The fresh concrete is poured into the molds and vibrated for about 1 min with a vibration table, then its surface is covered with a plastic film. After the first 24 h, the samples are demolded and cured in a climate chamber at a temperature of 20 °C and relative humidity of 95%, following ASTM C192-19 [41].

### 2.4. Test Methods

#### 2.4.1. Isothermal Calorimetry

The isothermal calorimetry tests are conducted with a TAM Air isothermal calorimeter (TA Instruments, New Castle, DE, United States) based on ASTM C1679-14 [42]. Following the mixes listed in Table 3, the pastes (without LWAs) are firstly mixed and vibrated for about 2 min. After adding coir fibers, they are vibrated for an additional 2 min. The mixed pastes with coir fibers are transferred into ampoules, and then the ampoules are loaded into the calorimeter. All measurements are conducted for 168 h (7 days) at a constant temperature of 20 °C. All mixes are measured twice to ensure the results.

#### 2.4.2. Water Permeable Porosity

The mass changes of LWAC under different conditions are evaluated by adopting the vacuum-saturation technique, following the method described in NT Build 492. The water-permeable porosity can be calculated from the measured mass changes:

(2) $$P_{v,\mathrm{water}}=\frac{m_{s}-m_{d}}{m_{s}-m_{w}}\times 100\%$$

where P_v,water_ is the water-permeable porosity (%), m_s_ is the mass of the saturated samples under surface-dry condition in the air (g), m_w_ is the mass of the saturated samples in the water (g), and m_d_ is the mass of the samples after oven-dried (g).

#### 2.4.3. Scanning Electron Microscope-Energy Dispersive X-Ray (SEM-EDX)

The ITZs between fibers and cement are observed by Scanning Electron Microscopy (SEM) and Energy Dispersive X-ray (EDX) spectroscopy with a Phenom Pro X SEM (Thermofisher, Waltham, MA, USA). After curing for 28 days, the specimens are cut into small fragments and are embedded in epoxy resin. Then the vacuum impregnation is conducted on all specimens. After that, the surface of samples is polished, then washed by an ultrasonic cleaner with absolute ethanol. Afterward, the samples are dried in an oven at 40 °C and then coated with gold. An accelerating voltage of 15 kV is applied for all samples.

#### 2.4.4. Densities

Samples of LWAC with a size of 160 mm × 40 mm × 40 mm are cast for density testing. The mass of samples is measured under both ambient and oven-dry conditions. Then the apparent density and dry density of LWAC are determined by calculating from the mass and size of the samples. At the age of 7 and 28 days, three samples for each mixture are tested to obtain the average density.

#### 2.4.5. Compressive Properties

The compressive strength of LWAC is measured under load control by an Automax 5 Automatic tester (Controls S.p.A., Milan, Italy), following ASTM C109 [43]. The loading speed used is 2400 N/s. Samples with a dimension of 40 mm × 40 mm × 40 mm are tested at the ages of 3, 7, and 28 days. At each age, six samples are measured for each mixture to obtain the average compressive strength.

#### 2.4.6. Flexural Properties

The flexural properties of LWAC are measured under three-points bending combined with displacement control with an Instron 5967 (Instron, Norwood, MA, United States) universal testing machine, following the ASTM C348 [44]. An un-notched specimen has been used with a span support of 100 mm. The mid-span deflection rate applied is 0.5 mm/min. Samples with a dimension of 160 mm × 40 mm × 40 mm are tested at the ages of 3, 7, and 28 days. At each age, three samples are measured for each mixture to determine the average flexural strength. The flexural strength can be determined from the ultimate load:

(3) $$\sigma =\frac{3\mathrm{FL}}{2{\mathrm{bh}}^{2}}$$

where F is the ultimate concentrated load, L is the span length; b and h are the width and height of samples.

The flexural toughness, expressing the energy absorption capacity of a material, can be calculated from the area under the load-deflection curves:

(4) $$T_{f}=\int_{0}^{\delta_{u}}F\left(\delta \right)d\delta$$

where δ_u_ is the maximum deflection, generally about 10 mm in this study.

#### 2.4.7. Drying and Autogenous Shrinkage

Both the drying and autogenous shrinkage tests are conducted on samples of 160 mm × 40 mm × 40 mm. After 24 h sealed curing, the specimens are demolded for shrinkage tests, and additionally, the specimens for autogenous shrinkage test are immediately sealed with a hydrophobic plastic film to avoid moisture loss. Then, samples for drying and autogenous shrinkages are exposed in an environmental chamber with a relative humidity of 50% and 99%, respectively. The zero-time of measurement is defined as the demolding time for both shrinkages. The length change along the longitudinal axis of samples is measured by a digital length comparator (±0.001 mm) at the desired ages. The initial length of all samples is straightway measured, then the followed length is measured once per working day until 28 days and three times a week until 56 days. Three specimens are tested for each mix to obtain the average values of the two shrinkages. All the tests have been carried out at a room temperature of 20 °C.

## 3. Results and Discussion

### 3.1. Reaction Kinetics

**Figure 5:**
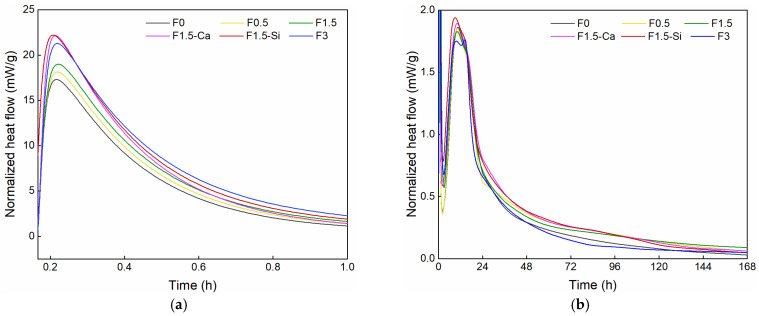
Normalized heat flow of cement paste with coir fibers of various contents and different treatments as a function of time, measured by isothermal calorimetry (**a**) within 1 h and (**b**) within 7 days.

The heat evolution curves of all mixes within the first 7 days are shown in Figure 5. Three main peaks are observed in samples with coir fibers, consistent with the plain cement hydration [45]. The first sharp peak is mainly corresponding to the dissolution of cement. Then a second peak related to C_3_S reaction appears, yielding CH and C–S–H. After this, a third peak concerning refreshed C_3_A hydration is observed. In all samples, the time of heat peaks shows no significant inhibition, although different intensities of peaks are presented. The heat evolution curves of samples with different coir fiber contents show more intensive first and third peaks, but a weaker second peak with increasing fiber amount. The increased first peak is mainly due to the coupling effect of different leached sugars from coir fibers leading to an acceleration of the dissolution of cement grains. Sugars prefer bonding to the positive ions in cement pore solution, especially Ca^2+^, by complexing or chelating because of their large amount of hydroxyl and aldehyde groups [46,47,48], which allows ions coexisting in solution at much higher concentrations without precipitation. This results in a promoted cement dissolution attributing to a higher ion concentration in the initial pore solution including sulfate, Al and Si. The second peak decreases because the higher initial ions concentration in cement inhibit the further reaction of C_3_S and C_2_S. On the other side, sugars adsorbed or complexed on cement grains or hydrated cement nuclei poison the nucleation sites of CH and C–S–H, thereby inhibiting their precipitation and further formation [49,50]. The third peak is promoted by the improved dissolution of SO_4_^2−^ and Al ions, yielding greatly refreshed AFt and AFm phase production [51,52,53]. More fibers leach out additional sugars which have a stronger influence on the reaction processes. Except for mechanisms above, the entrained internal water accelerates the cement hydration during the whole process, but it could be impaired by the leachate effects. Within samples with different pretreated coir fibers, all reaction processes are accelerated. Besides the effect of fiber leachates, Ca(OH)_2_ treatment compensates the Ca^2+^ inhibited by fiber leachates. It also increases the pH in the early-age pore solution, which facilitates hydration rate. Nano-silica treatment introduces both pozzolanic and size effects which can help hydration processes. The pozzolanic effect is caused by additional amorphous silica provided by the nano-silica solution, which reacts with calcium hydroxide forming additional C–S–H [54,55]. In addition, due to the small particle size of silica, greater nucleation sites are provided for C–S–H growth, which accelerates cement hydration [56].

It should be noted that after the main courses of hydration, the residual hydration rate of cement containing different coir fiber contents shows interesting trends after 1 day. The residual hydration rate of cement with 0.5% fibers remains at a higher level than that of the plain one within the 7 days. Simultaneously, the residual hydration rate of cement containing 1.5% fibers surpasses that of 0.5% fibers after 4 days, and it continues to be greater until the end of the test. Samples with 3% fibers show a higher growth rate of residual hydration, which exceeds the value of the reference one after about 6 days. It appears that the retardation period caused by fiber leachates ends gradually as if a retardation barrier is overcome. When the number of nucleation sites is more than the quantity of leached sugars occupied, future formed sites are no longer being poisoned [47]. After that, the hydration products can deposit at a higher rate since the excess amount of them is formed in previous courses. Furthermore, it reveals that 0.5% of fibers promote the early age hydration within 3 days, while higher contents of fibers benefit for later hydration. Besides, in a long period, the final hydration degree of cement could be improved by adding coir fibers. Therefore, an appropriate amount of leachates from coir fibers substantially acts as a delayed accelerator rather than a retarder, which causes part of cement hydration delayed, but then shows a higher residual rate of reaction [57].

### 3.2. Porosities and Microstructure

**Figure 6:**
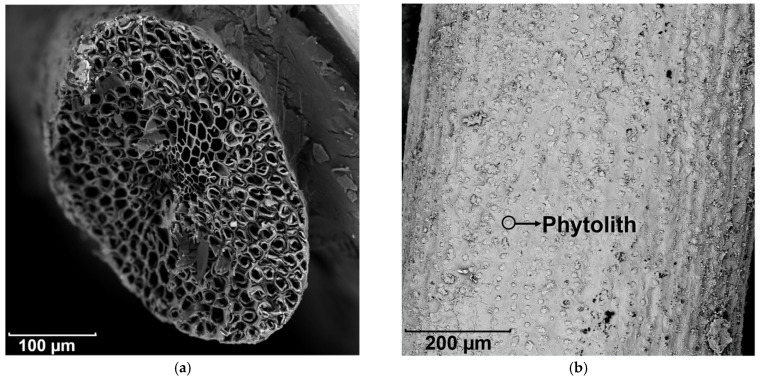

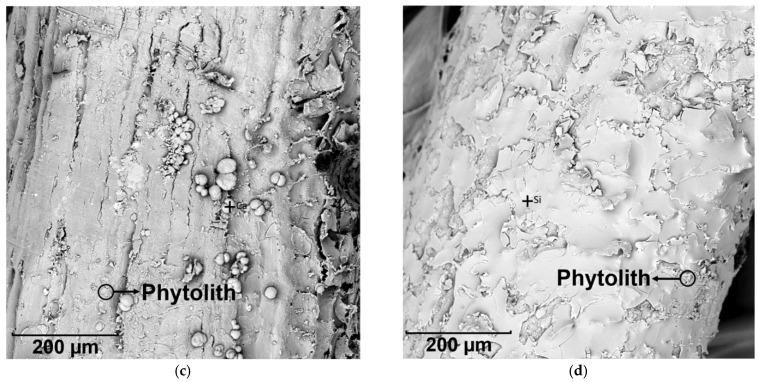
SEM pictures of coir fibers: (**a**) Cross-section of raw coir fibers, (**b**) surface morphology of raw coir fibers, (**c**) Ca(OH)_2_ treated fibers, and (**d**) nano-silica treated fibers.

The cross-section and surface morphology of raw coir fibers are presented in Figure 6a,b, respectively. Generally, coir fibers have a porous internal structure and wax covered external surface [58]. Additionally, amorphous silica bodies termed phytoliths are embedded in fiber surface [59]. The morphology of coir fibers pretreated with Ca(OH)_2_ and nano-silica are presented in Figure 6c,d, respectively. Ca(OH)_2_ treatment leads to the removal of the enveloped waxy layers, but the reservation of phytoliths, and an additional introduction of Ca deposited on the coir fibers. Nano-silica treatment results in a coating film of SiO_2_ on the fiber surface. Both treatments generate a rougher but a more homogeneous fiber surface, and maintain the phytoliths, which are expected to provide mechanical internal locking as anchors after reacting with cement [60].

**Figure 7:**
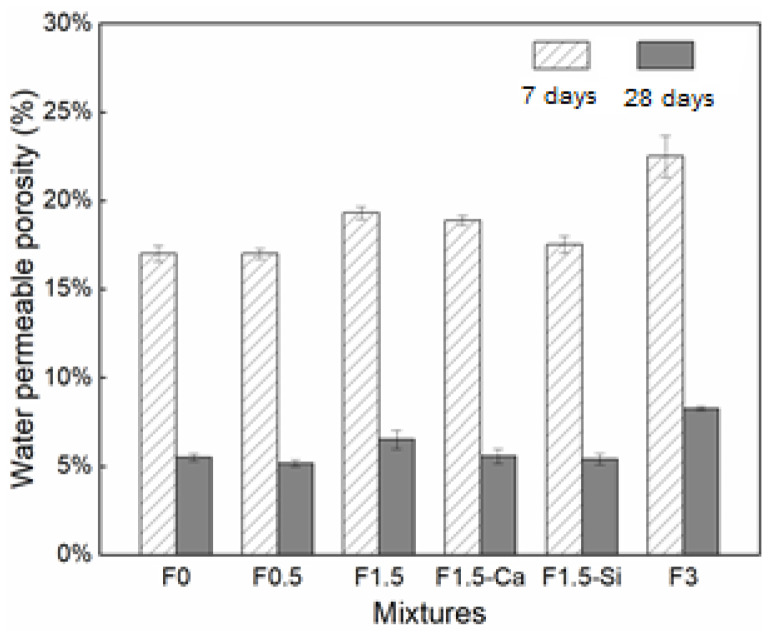
Water permeable porosity of LWAC with coir fibers of various contents and different treatments on 7 days and 28 days, calculated from the measured mass changes.

The water-permeable porosity of concrete with coir fibers of different dosages and treatments at 7 and 28 days are exhibited in Figure 7. The porosity of concrete decreases with increasing ages owing to the more adequate cement hydration. With a higher content of coir fibers, larger porosity is presented. This is due to the porous structure of coir fibers themselves, which attributes to more air (i.e., carried by fibers) and pores (i.e., contained in fibers) into the mixture. Therefore, more voids will be introduced into the concrete, contributing to a more porous structure. Whereas concrete with 0.5% fibers shows slightly less porosity as the reference, which is probably because of a denser matrix caused by a leached accelerator, compensating for the relatively low porosity created by low content of fibers. With pretreated fibers, the porosity of concrete decreases finely because the accelerated hydration caused by the treating agents reduce the pores of ITZs, as described in the following.

**Figure 8:**
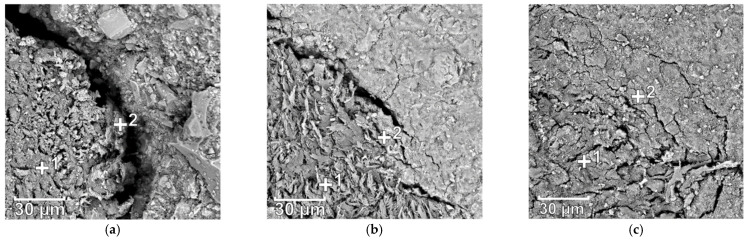
ITZs (Interfacial Transition Zones) with (**a**) raw coir fibers, (**b**) Ca(OH)_2_ treated fibers, and (**c**) Nano-silica treated fibers.

The SEM micrographs of ITZs between both raw and pretreated coir fibers and matrix are shown in Figure 8. It can be seen that fiber treatments have a positive effect on the ITZs. The ITZs of the raw fibers are usually porous, and cracking along the fibers can be observed. This is caused by the heterogeneity of fibers and cement resulting in weak ITZs, which are in favor of cracks propagating. Another explanation can be the desiccation shrinkage of fibers since fibers are saturated with water which may be taken up by cement for further hydration [25,61]. However, the ITZs formed with the pretreated fibers are much denser and less cracked, and a better solid bonding can be seen. It is because these treatment accelerators attached to fibers contribute to more hydration products precipitated on the interfaces, which upgrades the contact between fibers and matrix. Additionally, for the samples with nano-silica treated fibers, the most condensed ITZs are presented. It is caused by its nano-scale causing filling action, which allows the hydration products to fill the voids of the general C–S–H structure [62].

**Figure 9:**
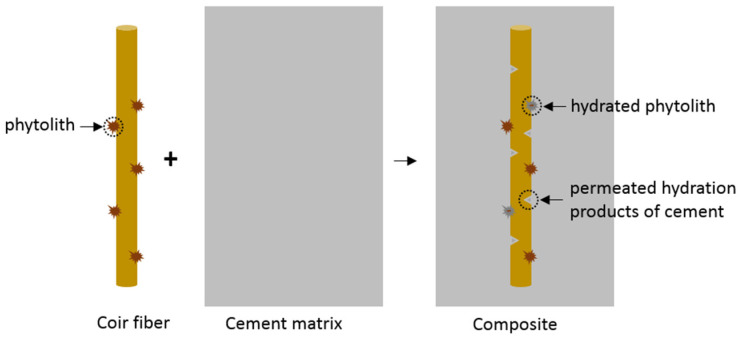
Schematic diagram of the interlocking effect between coir fibers and cement matrix.

The EDX analysis of elemental compositions near coir fibers is presented in Table 4. These spots of ref-1 and ref-2, F-Ca-1 and F-Ca-2, as well as F-Si-1 and F-Si-2 in Table 4 are corresponding to the indicated points 1 and 2 in Figure 8a–c, respectively. In addition, both Ca/Carbon and Si/Carbon ratios are calculated to characterize the C–S–H amount, and these two ratios are both at a higher value, indicating a greater C–S–H amount. A high Ca concentration is observed on the surface of raw fibers, and a considerable Ca is also observed inside of fibers, which demonstrates the chelating Ca^2+^ effect of untreated fibers. Moreover, less Si can be found on the interface, and no Si is observed in fibers, which represents less C–S–H on the fiber surface and no C–S–H infiltrated into fibers, leading to poor interfaces between fibers and matrix. For both ITZs of the pretreated fibers, both increased Ca/Carbon and Si/Carbon ratios are shown on the surface and interior of fibers, indicating a higher accumulation of hydration products [63,64]. The permeated hydration products form anchorages within fibers, as shown in Figure 9. It leads to enhanced mechanical interlocking and entanglement between fibers and matrix, therefore, coir fibers could bond better to cement, providing better performance.

### 3.3. Drying and Autogenous Shrinkages

**Figure 10:**
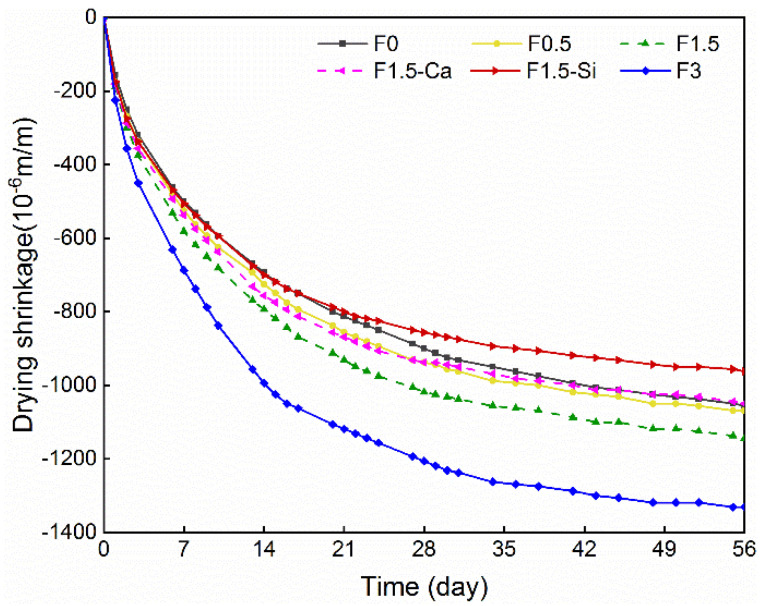
Drying shrinkage of LWAC with coir fibers of various contents and different treatments measured in 56 days.

Figure 10 shows the drying shrinkage development of concrete with coir fibers within 56 days. The drying shrinkage increases rapidly within the first two weeks, after which its growth rate slows down since less water loss occurs in the later age. Moreover, the drying shrinkage notably rises by the increased fiber contents. This behavior can be firstly explained by the increased water introduced by more coir fibers in the system, which results in a higher amount of evaporated water loss, thus causing larger desiccative deformation. Another explanation is that coir fibers enlarge the porosity in the matrix and increase the connectivity of pores [65,66], which accelerates the drying process and consequently increases the drying shrinkage. Additionally, concrete with pretreated coir fibers shows slightly lower drying shrinkage as compared to the one with untreated fibers. It is because the pretreated fibers can promote the cement hydration, leading to considerably less water remained in concrete, and thus lower drying shrinkage is obtained.

**Figure 11:**
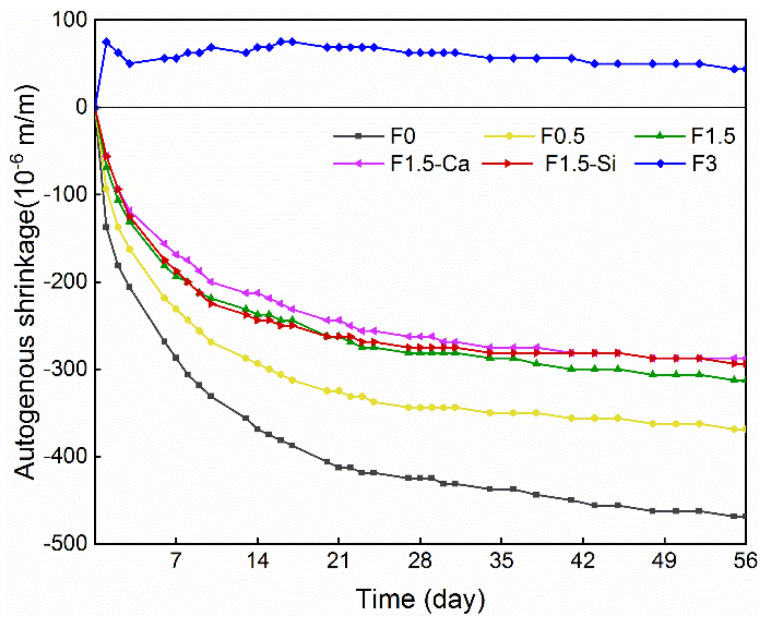
Autogenous shrinkage of LWAC with coir fibers of various contents and different treatments measured in 56 days.

Figure 11 shows the autogenous shrinkage development in coir fibers reinforced concrete within 56 days. Most of the autogenous shrinkage is developed within the first week and then its intensity appears to reduce because of subdued cement hydration. Coir fibers result in a reduction in the autogenous shrinkage, and the increasing amounts of fibers significantly decreases the autogenous shrinkage. This performance is due to the higher quantity of coir fibers contributing to sufficient internal curing water, which compensates for cement internal self-desiccation caused by chemical hydration [67]. As a result, a lower capillary pressure is induced, and thus less autogenous shrinkage is observable. Another reason is that the delayed hydration caused by fiber leachates leads to suppressed development of autogenous shrinkage. Furthermore, fibers can sustain tensile strain and restrain cracking introduced by drying, and thus decrease the autogenous shrinkage of concrete. When the fiber content is set to 3%, the concrete exhibits a rapid expansion during the first day and continues showing a slight expansion during the whole period, therefore, the autogenous shrinkage of concrete is completely eliminated. This expansion is mostly because of the higher crystallization stress resulting from the increasing level of the oversaturated portlandite in solution caused by fiber leachates [68], which can entirely overwhelm the effect of self-desiccation, resulting in almost no autogenous shrinkage. Therefore, when plenty of coir fibers are used, sufficient water can be introduced for internal curing in the cementitious matrix. Additionally, the pretreated coir fibers promote cement hydration resulting in higher autogenous shrinkage, but the improved ITZs benefit for reducing this shrinkage, therefore, a slightly reduced autogenous shrinkage can be seen.

### 3.4. Compressive Strength

**Figure 12:**
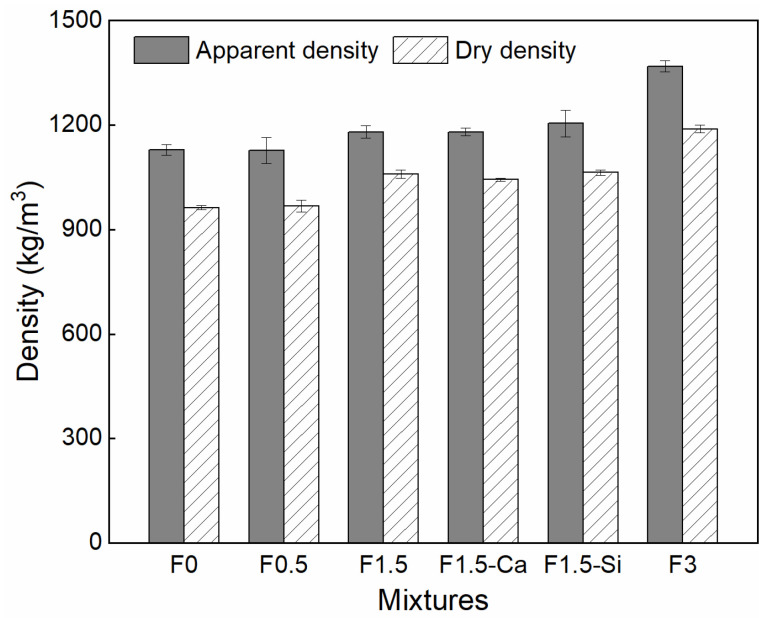
Apparent and dry densities of LWAC with coir fibers of various contents and different treatments.

**Figure 13:**
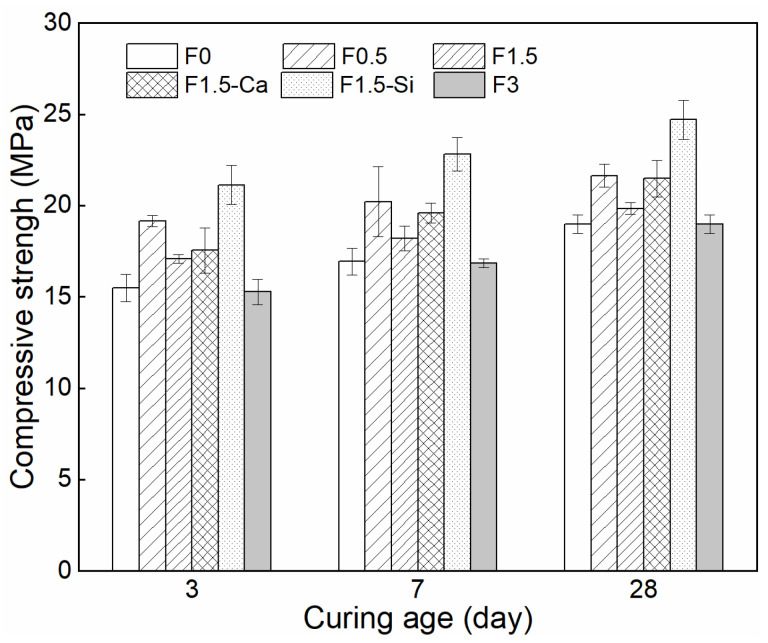
Compressive strength of LWAC with coir fibers of various contents and different treatments measured at 3, 7, and 28 days.

The apparent and dry densities of LWAC with coir fibers are presented in Figure 12. The apparent density of concrete is within the range of 1127 and 1369 kg/m^3^, and the dry density is between 960 and 1179 kg/m^3^, which meets the requirement of light-weight concrete. The 3, 7, and 28 days compressive strengths of all mixtures are depicted in Figure 13. The compressive strength of coir fibers reinforced LWAC increases while aging, but presents different development rates. Among the samples with different coir fiber contents, samples with 0.5% fibers give the faster development of early-age strength within 3 days, while the samples with 1.5% and 3% fibers show a higher increasing rate of later strength. For samples with different pretreated coir fibers, nano-silica treatment is more effective in enhancing the early 3-day strength than Ca(OH)_2_. It is in agreement with the previous hydration results, since cement hydration is positively related to the corresponding strength. Therefore, cement hydration promoted by coir fibers and treatments at certain ages consequently leads to the increased strength.

The compressive strength of LWAC slightly increases by adding coir fibers, while it decreases with the increased coir fiber content. The 28-day compressive strength of the reference sample is 19.0 MPa, and those of mixtures with 0.5% and 1.5% coir fibers are 21.6 MPa and 19.8 MPa, respectively, corresponding to an approximately 14%, and 5% increase. The reasons behind this phenomenon are that coir fibers can promote hydration and can also create a bridging effect. Comparably, a low content of fibers added into cement results in a proper amount of leachates which could promote cement hydration [57], contributing to forming a much denser matrix. Furthermore, coir fibers retard the cracks propagation and bridge the matrix from rupture under compression, thus a larger force is required for concrete failure [69,70].

However, when the coir fiber content further increases to 3%, no improvement is observed in the compressive strength. The explanations seem to be related to the mixing procedure, the porous structure of fibers, and the introduced ITZs between the fibers and matrix. More fibers tend to cause fiber agglomeration during mixing, and thus has a negative effect on the compaction and homogeneity of the mixture [71,72], leading to reduced bonding between the fibers and matrix and increased flaws in the mixture. In addition, due to the cellular structure of coir fibers, a higher fiber content leads to larger porosity in the mixture. Moreover, more weak ITZs are introduced which are easily subject to failure. Finally, the combined action from these factors deteriorates the compressive strength of the mixture.

Besides, when fiber content is set at 1.5%, both treatments for coir fibers increase the 28-day compressive strength of concrete considerably. It shows 21.5 MPa and 24.7 MPa with the treatment of Ca(OH)_2_ and nano-silica, indicating about 13% and 30% increase as compared to the reference one and about 8% and 25% rise as compared to the sample with 1.5% untreated fibers, respectively. The underlying reason is that treatments cause more hydrated cement around coir fibers, which leads to improved ITZs and enhanced mechanical anchoring between the fibers and matrix, as explained in Section 3.2, ensuring better performance of the concrete.

### 3.5. Flexural Properties

**Figure 14:**
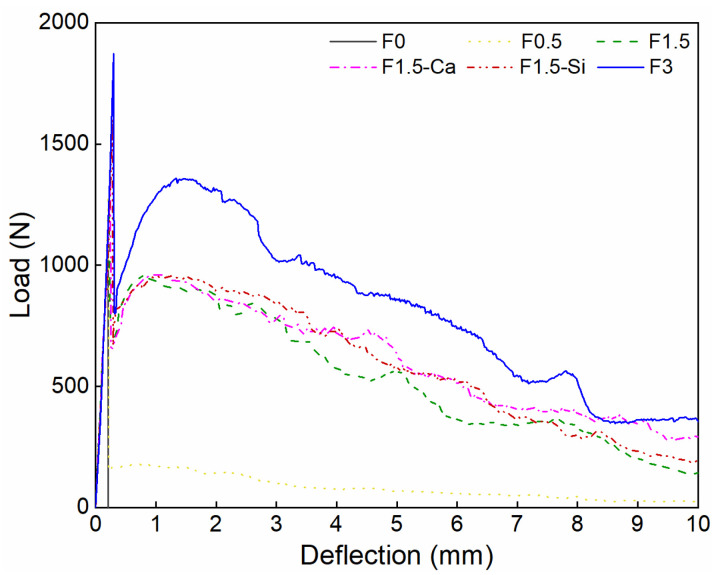
Average load-deflection curve of LWAC with coir fibers of various contents and different treatments.

**Figure 15:**
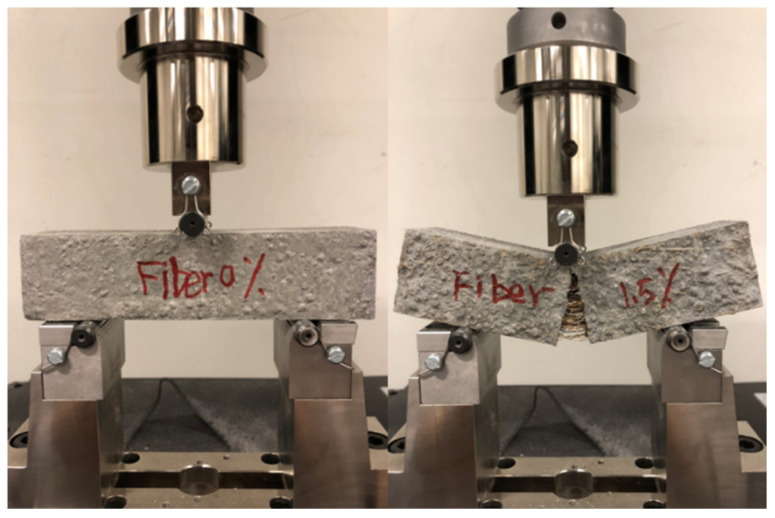
Flexural fracture characteristic of LWAC. The left sample without coir fibers and the right one with 1.5% coir fibers.

The load-deflection curves are shown in Figure 14. The initial cracking strength significantly increases with improved fiber contents and fiber treatments. After the initial cracking, a sharp drop in loads in all samples can be seen because of the reduced load-bearing capacities. Usually, a residual stress is maintained in the samples with fibers, while in the reference samples, the stress drops to zero directly. The flexural fracture characteristic is shown in Figure 15. In the left sample without coir fibers, a penetrative crack rapidly occurs in the middle of the sample, and no deformations are seen. While in the right one with coir fibers, the cracking gradually develops and a clearly deformation is presented. These phenomena are caused by the bridging effect of coir fibers as drawn from the research of both steel and synthetic fibers [18,73,74]. In addition, samples with a higher fiber content or treated fibers show a lower drop of loads. This is because of more active fibers or better bonding behaviors, allowing improved tolerance to stress, and thus higher residual strengths can be observed [19,75].

**Figure 16:**
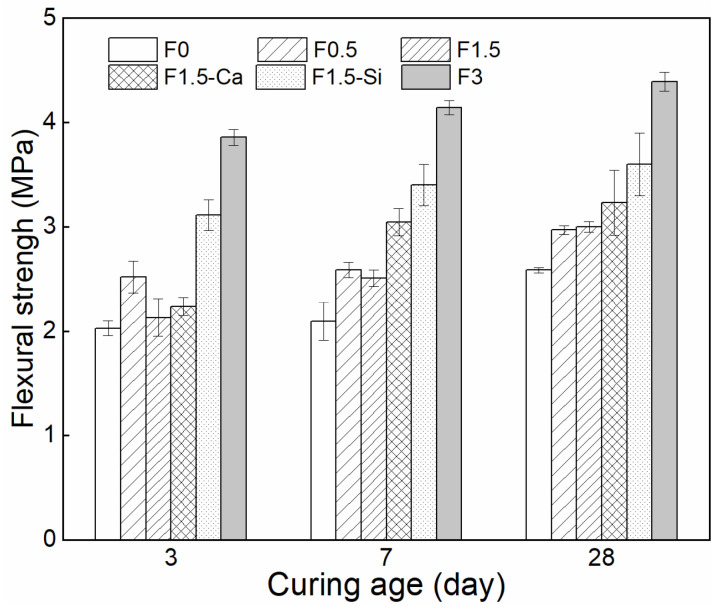
Flexural strength of LWAC with coir fibers of various contents and different treatments measured at 3, 7, and 28 days.

The 3, 7, and 28 days flexural strengths of each of the mixtures are presented in Figure 16. The flexural strength development of LWAC is consistent with its compressive strength, i.e., samples with 0.5% fiber content and nano-silica pretreated fiber are beneficial for early strength. Conversely, the flexural strength of LWAC increases with increased fiber content. The highest 28-day flexural strength of the samples with 3% fibers is about 4 MPa, indicating an increase of about 70% as compared to the reference samples (2.6 MPa). The main explanation can be that a load applied can be transferred to coir fibers which are more tolerant to tension than cement matrix [18,19,76], and more fibers can bear higher tensile stress for cement, resulting in a higher flexural strength. The flexural strength of the samples with 1.5% fibers shows a negligible improvement compared to 0.5% fibers, since fibers can introduce additional defects into the matrix. Moreover, both treatments slightly increase the 28-day flexural strength of concrete, and the nano-silica treatment presents a better strengthening effect because of its much improved ITZs.

**Figure 17:**
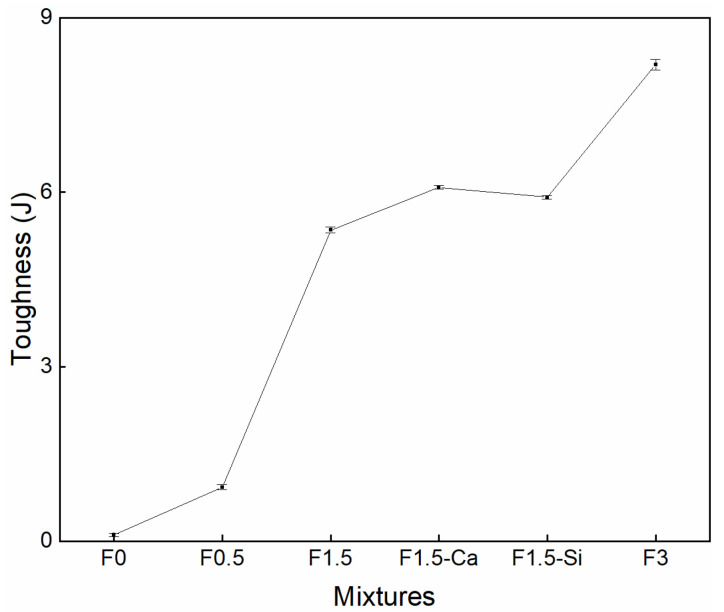
Flexural toughness curve of LWAC with coir fibers of various contents and different treatments at 28 days.

The flexural toughness of each mixture at 28 days is shown in Figure 17. The toughness is significantly enhanced by increasing the amount of fibers. The toughness of the samples with 3% fibers shows about an 8 times increase from the reference samples. This is because the cracks extending acquires stretching, debonding, and breaking of fibers [71,75,77], during which it generates a considerable energy loss, contributing to increased fracture toughness. The toughness is slightly increased because of fiber treatments, which reveals that fiber content has a greater influence than treatments on the toughness. Even though fiber treatments can improve the ITZs between the fibers and cement matrix, treatments can make fibers stiffer and more brittle [78,79], thus reducing the energy absorption ability. Therefore, the total energy consumption of concrete with pretreated fibers presents minimal difference compared with non-treated fibers.

### 3.6. Reinforcement Mechanisms of Coir Fibers

Here, there are three mechanisms that can be used to explain the reinforcement effects of saturated raw coir fibers, which are hydration accelerating, internal curing, and mechanical bridging. Besides, both strengthening and weakening effects are presented as the result of adding fibers, which can be balanced by applying an optimum fiber amount. Firstly, coir fibers in cement can leach out sugars, and their coupling effect could chemically accelerate the cement hydration resulting in more saturated hydration, thus improving the mechanical properties. Nevertheless, a surplus of fibers can leach additional sugars, which can inhibit the cement hydration for a long duration. Secondly, saturated fibers supply extra water for internal curing, therefore, autogenous shrinkage decreases. Less micro-cracks occur in the interior of concrete, leading to ameliorative macro-mechanical properties. Finally, similar to artificial fibers like steel and synthetic fibers, coir fibers can reinforce concrete by physically bridging. Coir fibers can carry tensile stress and absorb fracture energy to protect concrete from damage, contributing to increased compressive strength, flexural strength, and fracture toughness. However, an excess amount of fibers (3 wt.% in this study) can cause adverse effects on the compressive strength of concrete because of introduced defects from processing procedures like fiber clustering and weaker ITZs.

Moreover, treatments of coir fibers can also enhance the mechanical properties of concrete. On one hand, the treating agents at the surface of coir fibers accelerate the hydration process close to the fibers, and thus more hydration products precipitated on the fiber surface, leading to better ITZs and improved bonding between fibers and matrix. On the other hand, more hydration products permeate into the fibers, forming anchors to improve mechanical interlocking and contact between these two phases. Therefore, coir fibers with treatments have better bonds to the cement matrix, leading to improved performance of concrete.

## 4. Conclusions

This paper presents the influences of coir fibers on the hydration reaction, shrinkage performance, and mechanical properties of cement-based LWAC. Furthermore, two treatments, namely Ca(OH)_2_ and nano-silica impregnation, have been employed to further improve the above properties of concrete. Finally, potential mechanisms contributing to coir fibers reinforcement are discussed. Based on the results obtained, the following conclusions can be drawn:

- The leached sugars from coir fibers with the amount ranging from 0.5% to 3% act as a delayed accelerator rather than a retarder, which promotes cement hydration in the long term.
- With the increased fiber content up to 3%, the drying shrinkage of concrete increases while the autogenous shrinkage decreases. When a sufficient content of coir fibers, i.e., 3% in this study is applied, fibers can be used for internal curing.
- The compressive strength of LWAC increases slightly by adding fibers, and the maximum increase is in the order of 14% in samples with 0.5% coir fibers as compared to the reference. As the coir fiber content increases from 0% to 3%, the flexural strength and toughness show a maximum increase in the order of 70% and 800%, respectively.

Treatments of coir fibers promote the hydration reaction of cement due to the accelerating effect of the different treating agents, i.e., Ca(OH)_2_ and nano-silica, used in this study. Ca(OH)_2_ treated fibers and nano-silica treated fibers increase the 28-day compressive strength of LWAC in the order of 13% and 30%, respectively, while slightly influencing the 28-day flexural strength and toughness. Three underlying mechanisms are discussed to comprehensively interpret the coir fibers reinforcement, including hydration accelerating, internal curing, and mechanical bridging. Therefore, it can be concluded that coir fibers can be used to replace some elastic synthetic fibers to effectively reinforce concrete, by also providing additional positive effects such as hydration promotion and internal curing. However, besides the mechanical properties, the durability of this coir fibers reinforced concrete is a vital factor for application. Further investigation on the durability of concrete with coir fibers and coir fibers themselves is needed and will be a subject for our future research.

## Figures and Tables

**Table 1:** Physical and chemical properties of coir fiber.

Bulk Density (kg/m^3^)	Specific Density (kg/m^3^)	Average Length (mm)	Average Diameter (µm)	Average Tensile Strength (MPa)	Cellulose (wt.%) [37]	Hemicellulose (wt.%) [37]	Lignin (wt.%) [37]
69.8	1539.6	15	250 ± 42	400 ± 83	36.6 ± 0.15	37.0 ± 0.15	22.2 ± 0.05

**Table 2:** Sugars concentration in the leachates from coir fibers.

Sugars	Concentration (mg/mL)
Arabinose	0.21 ± 0.02
Galactose	0.08 ± 0.01
Glucose	0.04 ± 0.01
Xylose	0.56 ± 0.05
Mannose	0.02 ± 0.01
Galacturonic acid	0.02
Glucuronic acid	0.07 ± 0.01
Total sugars	0.91 ± 0.1

**Table 3:** Mix design of light-weight aggregate concrete (LWAC).

No.	Cement (kg/m^3^)	LWA-1 (kg/m^3^)	LWA-2 (kg/m^3^)	LWA-3 (kg/m^3^)	LWA-4 (kg/m^3^)	LWA-5 (kg/m^3^)	Water (kg/m^3^)	SP (wt.%)	Coir Fibers (wt.%)
F0 (Ref)	526.7	85.1	32.3	41.2	43.9	60.9	210.7	0.8	0.0%
F0.5	524.1	84.6	32.1	41.0	43.7	60.6	209.6	1.0	0.5%
F1.5	518.8	83.8	31.8	40.6	43.3	60.0	207.5	2.3	1.5%
F1.5-Ca	518.8	83.8	31.8	40.6	43.3	60.0	207.5	2.3	1.5%
F1.5-Si	518.8	83.8	31.8	40.6	43.3	60.0	207.5	2.3	1.5%
F3	510.9	82.5	31.3	40.0	42.6	59.1	204.4	4.1	3.0%

**Table 4:** Chemical compositions around ITZs. The spots corresponding to the specified regions in Figure 8.

Treatments	Spots	Chemical Elements (wt.%)				
		Carbon	Ca	Si	Ca/Carbon	Si/Carbon
Reference, no treated	ref-1	55%	9%	0%	0.16	0
	ref-2	17%	40%	6%	2.33	0.35
Ca(OH)_2_ treated	F-Ca-1	44%	10%	3%	0.22	0.06
	F-Ca-2	14%	19%	8%	1.37	0.55
Nano-silica treated	F-Si-1	33%	19%	34%	0.59	1.03
	F-Si-2	11%	52%	40%	4.99	3.79

## Data Availability

Data available on request due to restrictions eg privacy or ethical.
